# Exploring the interplay between diet, obesity, mental health, and the gut microbiota: the MIND-GUT intervention study, study protocol

**DOI:** 10.3389/fnut.2025.1703255

**Published:** 2025-12-10

**Authors:** Deborah Gustafson, Elisabet Rothenberg, Steinn Steingrimsson, Hanne Krage Carlsen, Fabrizio Belloni, Nagalakshmi Eruvuri, Rajna Knez, Erika Olsson, Robert D. Burk, Hellas Cena, Rachele De Giuseppe, Gianluca Tognon

**Affiliations:** 1 Department of Neurology, SUNY Downstate Health Sciences University, Brooklyn, NY, United States; 2 Department of Nursing and Integrated Health Sciences, Kristianstad University, Kristianstad, Sweden; 3 Department of Psychiatry for Affective Disorders, Region Västra Götaland, Sahlgrenska University Hospital, Gothenburg, Sweden; 4 Section of Psychiatry and Neurochemistry, Institute of Neuroscience and Physiology, Sahlgrenska Academy, University of Gothenburg, Gothenburg, Sweden; 5 School of Health Sciences, University of Skövde, Skövde, Sweden; 6 Department of Surgical Sciences, Uppsala University, Uppsala, Sweden; 7 Departments of Pediatrics, Microbiology & Immunology, Epidemiology & Population Health, and Obstetrics, Gynecology & Women's Health, Albert Einstein College of Medicine, Bronx, NY, United States; 8 Laboratory of Dietetics and Clinical Nutrition, Department of Public Health, Experimental and Forensic Medicine, University of Pavia, Pavia, Italy; 9 Unit of Clinical Nutrition, ICS Maugeri IRCCS, Pavia, Italy

**Keywords:** obesity, depression, anxiety, gut microbiota, MIND diet, mental wellbeing, BMI

## Abstract

**Background:**

Obesity and mental health disorders often co-occur, contributing to individual suffering and healthcare costs. Diet plays a key role in both weight management and mental wellbeing and may influence these through its impact on the gut microbiota. However, the mechanisms linking diet, obesity, and mental health remain insufficiently understood.

**Aim:**

We propose to conduct the MIND-GUT study in Sweden to evaluate feasibility as primary outcome (retention, adherence, and acceptability) of a 12-week dietary intervention based on the MIND diet and, as secondary outcomes, the influence of the latter on weight loss and mental wellbeing in adults with overweight and obesity [body mass index (BMI) ≥30 kg/m^2^]. The study will also explore changes in gut microbiota composition and diversity.

**Methods:**

In this randomized trial, 126 adults with obesity will be assigned to either an intervention group following a MIND diet or a control group receiving general dietary advice. Energy intake targets will be <2,300 kcal/day for men and <1,900 kcal/day for women. The primary outcomes are retention, adherence, and intervention acceptability (the latter assessed through qualitative interviews). Secondary outcomes include changes in body weight, BMI, waist and hip circumferences, fat mass, and mental health including perceived stress, depression, and anxiety symptoms. Gut microbiota diversity and composition will be analyzed from stool samples collected at baseline and 12-week follow-up.

**Discussion:**

This study will offer valuable insights into the relationship between diet, mental health, obesity, and the gut microbiota. Results will inform the design of future large-scale trials assessing the MIND diet's potential as a strategy for improving physical and mental health.

## 1 Introduction

### 1.1 Background and rationale

The coexistence of obesity and mental health problems is common, although there are still uncertainties regarding the underlying causal pathways (1). Both obesity and depression have high prevalence worldwide, representing a considerable burden for healthcare systems. In Sweden, more than half of the adult population is overweight or obese, with a rising trend over time (2). From 2006 to 2022, the proportion of adults aged 16–84 years (y) who were overweight or obese increased from 46 to 51%. The most significant increase observed in Sweden was among those aged 16–29 years (y), rising from 22 to 28%. Overweight and obesity prevalence increases with age and reached 62% among those aged 45–64 years in 2022. Differences were observed based on sex, education, and country of birth (2). More specifically, overweight or obesity was more common in men (56%) than in women (45%), although obesity prevalence was 16% in both men and women. Obesity was more common among those with only pre-secondary education, but the incidence is increasing in all education groups.

Therefore, it is strategic to identify dietary interventions that can reduce the risk and prevalence of obesity and potentially also have beneficial influences on mental health, including depressive symptoms (3). Weight loss as a result of dietary interventions has been shown to improve mood scores in individuals with obesity who also have clinical or sub-clinical depression (4). Emerging research outlines how diet modulates the gut microbiota and obesity risk, offering a foundation upon which mental health pathways may operate, though explicit links to mental wellbeing remain underexplored in humans (5). Clarifying these interrelationships could reveal why some individuals succeed while others struggle with weight loss. The primary objective of this study is to assess the feasibility of a dietary intervention based on the MIND diet in terms of retention and adherence (6). The secondary aim is to compare changes in body weight, BMI, mental health, and gut microbiota composition between the intervention and control groups.

## 2 Methods and analysis

### 2.1 Study design

This study is a 12-week randomized clinical trial comprising two study arms: the intervention and control groups.

### 2.2 Study setting and recruitment

We propose to recruit 126 adults with obesity, aiming for an approximately equal representation of men and women. The sample size was calculated considering a potential dropout of 10%, an expected 5% change in body weight and psychometric scores at follow-up (standard deviation 10%) and an 80% power to obtain a statistically significant result at *p* < 0.10. A threshold of 0.10 instead of 0.05 was chosen due to the exploratory nature of this trial. All study materials (invitation letters, consent form, and questionnaires) are included in the Supplementary material. This sample size is based primarily on feasibility and obtaining robust parameter estimates for a future larger (potentially multi-country) study.

The recruitment will be conducted in Skövde, Gothenburg, and nearby towns in the Västra Götaland Region in Sweden, primarily via primary health clinics and social media.

The inclusion criteria are:

- Men and women aged 25–50 years old.
- BMI ≥30 kg/m^2^. This threshold may be lowered to BMI ≥27 kg/m^2^ if recruitment proves challenging, considering that individuals with high BMI are frequently unavailable due to being treated by obesity clinics or using weight-loss medications.
- Agreement to participate in all aspects of the study protocol.
- Reliable access to the internet over the intervention period.
- Possession of an active email account for study-related communications.

Exclusion criteria include:

- Living with another study participant.
- Use of weight loss medication, antibiotics (last 6 months) or psychiatric medications (intakes >12 months will be accepted).
- Dietary restrictions incompatible with the intervention or the control group (e.g., vegetarians or vegans, whereas lactose- and gluten-intolerant individuals are admitted, and also individuals who only excluded meat only).
- Diagnosis of an eating disorder, diabetes, or polycystic ovary syndrome.
- Sensory deficits, e.g., loss of taste or smell.
- Participation in another intervention study.
- Inability to understand written and spoken English or Swedish.
- Pregnancy, lactation, or plans to become pregnant during the study period.

The age range of 25–50 years was selected to focus on individuals with established obesity at a life stage relevant to early-to-mid career stress and the onset of chronic lifestyle diseases. Foreign-born will be included and birth place data will be collected. This cohort is deemed most appropriate for investigating the specific neurocognitive and gut microbiota effects central to the MIND-GUT study's hypotheses while ensuring optimal feasibility and high compliance throughout the 12-week intervention.

Informed consent will be obtained after participants are provided with detailed information about the study, including risks and benefits, study procedures, the expected duration of participation, and the confidentiality measures in place to protect their privacy. Participants will have the right to withdraw from the study at any time without any consequences or penalty. We will ensure the confidentiality of all participants and their data by utilizing secure electronic data management systems and implementing robust data protection protocols. All personal data collected will be stored securely in accordance with the General Data Protection Regulation (GDPR) and Swedish data protection regulations.

This study has been approved by the Swedish Ethical Authority (Dnr 2023-05478-01). Any protocol amendments increasing participant risk will be submitted to the ethical authority for approval.

### 2.3 Intervention

#### 2.3.1 Data collection and management

All participants provide informed consent for participation and meet a qualified member of the research team (a dietician, a research nurse, or a nutrition researcher with clinical experience) at enrollment and 12 weeks to measure body weight (kg), body height (m), percentage of fat mass, body circumference (cm), hip and waist circumferences (cm), systolic and diastolic blood pressures (mmHg), and heart rate. The staff conducting these measurements are standardized and trained in blood pressure measure, and in the use of the calibrated Tanita (model BC545N) scale, which provides a hand-to-foot impedance measurement and body weight. Questionnaires include the main study questionnaire, querying sex assigned at birth, gender, date of birth, body image rating scales [Stunkard Scale (7)], number of usual sleep hours per 24 h, physical activity (based on the WHO GPAQ questionnaire), education, marital status, and smoking status (ever/never/current and number of cigarettes or e-cigarettes used per day), use of chewing tobacco (in Sweden called “snus”), use of pharmaceutical drugs, and current health conditions. The date of the last menstrual period will be queried for females. Other questionnaires will include: a questionnaire on gut health and bowel movement, a food checklist to inquire adherence to the MIND diet (8), the Perceived Stress Scale 4 (PSS-4) (9), to assess perceived stress, the Participant Health Questionnaire 9 (PHQ-9) (10) to assess depressive symptoms, the General Anxiety Disorder-7 (GAD-7) (11) for anxiety symptoms, and the Eating Attitudes Test [EAT-26 (12)]. All questionnaires will be administered online via “EvaSys” available on Skövde University's server, at baseline and 12 weeks, with the exception of the diet and gut health questionnaires which will also be filled out at 6 weeks.

**Figure 1:**
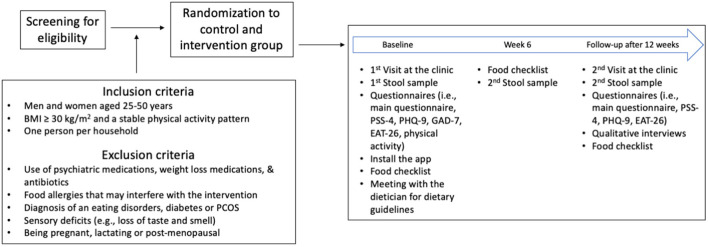
Detailed overview of the study protocol for intervention and control groups. BMI, body mass index; PCOS, polycystic ovary syndrome; PSS-4, perceived stress scale 4; PHQ-9, participants health questionnaire 9; GAD-7, general anxiety disorder-7; EAT-26, eating attitudes test.

At the conclusion of the intervention, participants will be asked to provide feedback on their experience and acceptance of the study through a qualitative, semi-structured interview. Participants will be invited to reflect on their experience with the diet, including adherence, challenges, satisfaction with dietary recommendations, and support received from staff, family members or other personal acquaintances. They may share feedback on the guidance provided, which includes periodical informational newsletters, reminders (e.g., for sample collection), and direct staff consultation to address adherence issues, side effects of the diet, and practical concerns, and suggest improvements. To promote participant retention and ensure complete follow-up, we will maintain regular contact through reminders and offer flexible scheduling options. Figure 1 provides a summary of the study process, while Table 1 depicts the schedule of enrollment, interventions, and assessments.

**Table 1:** Schedule of enrolment, interventions, and assessments.

	**Study period**				
	**Enrolment**	**Allocation**	**Post-allocation**		**Close-out**
TIMEPOINT	-t_1_	0	t_1(baseline)_	t_2(6weeks)_	t_3(12weeks)_
**ENROLMENT:**					
Eligibility screen	x				
Informed consent	x				
Allocation		x			
**INTERVENTIONS:**					
MIND Diet			x		
Control Diet			x		
**ASSESSMENTS:**					
Body weight			x		x
Body height			x		x
Body composition			x		x
Blood pressure			x		x
Heart rate			x		x
Stool sample			x	x	x
Main questionnaire			x		x
Diet checklist			x	x	x
Gut health questionnaire			x	x	x
GAD-7 questionnaire			x		x
PHQ-9 questionnaire			x		x
PSS-4 questionnaire			x		x
EAT-26 questionnaire			x		x
Qualitative interview					x

PSS-4, perceived stress scale 4; PHQ-9, participants health questionnaire; GAD-7, general anxiety disorder-7; EAT-26, eating attitudes test.

#### 2.3.2 The dietary intervention

Participants will be blinded as to their assignment. Unblinding is permissible only in medical emergencies when knowledge of the allocated intervention is essential for participant safety; in such cases, the allocation will be revealed through a secure procedure documented in the trial protocol. The intervention group will follow a standardized diet explicitly developed for this study, emphasizing principles of the MIND diet (see Table 2 for the list of recommended intakes, e.g., 100 g of green leafy vegetables at both meals, 50 g of berries at least 5 days a week), which includes foods potentially beneficial for mental health, such as those rich in omega-3 fatty acids and berries. Energy intake goals for both groups will be set at < 2,300 kcal (9,623.2 kJoules)/day for men and < 1,900 kcal (7,949.6 kJoules) per day for women, as recommended by the United States National Research Council (13). This approach aims to control for the potential confounding effects of weight loss on mental health outcomes. The meal plan will suggest five meals per day (i.e., breakfast, midmorning snack, lunch, afternoon snack, and dinner). Consumption frequencies of each food group are presented in Supplementary material.

**Table 2:** List of foods included in the intervention diet.

**Foods encouraged in the MIND diet^a^**	**Recommended servings**
Green leafy vegetables	100 g at both lunch and dinner
Other vegetables	100 g at both lunch and dinner
Nuts and seeds	At least 20 g/day
Berries	50 g at least 5 days/week
Other fruits	2 servings a day
Beans/Legumes	At least 3 servings/week
Whole grains	3 servings/day
Fish (not fried)	2–3 servings/week
Poultry (not fried, white meat/skinless)	2 servings/week
15.6-2.2,-1.3242ptExtra virgin olive oil	2–3 tablespoons per day
**Foods to limit in the MIND DIET** ^a^	
Grains (with no specification whether refined or not)	Not included
Red and processed meats	Max 1 servings/week
Butter and margarine	Avoid
Oil (with no specification about the type)	Not included
Cheese (whole fat)	1 portion per week
Cheese (low fat)	1–2 portions per week
Desserts	Not included
Fried foods and fast foods	Not included

Both lunch and dinner will include a main dish, vegetables as a side dish, and a piece of fruit. Participants in the intervention diet group will be allowed to replace a main dish (e.g., a recipe or a generic meal item like 2 eggs or a piece of cheese) only with another dish from the same food group. Breakfast and snacks. Participants in the intervention group will be instructed to limit their salt intakes, whereas no such indication will be given to participants in the control group.

^a^Liu et al. (6) and Arjmand et al. (15).

The control group will receive general guidance, providing flexible advice for building balanced meals in line with the current Swedish national recommendations. Their support materials, including a recipe book, reflect traditional Swedish healthy eating patterns and promote overall balance and variety. Critically, these materials do not specifically emphasize or systematically increase the target components of the MIND diet. The intervention group will receive a standardized diet and a recipe book explicitly developed to maximize adherence to the MIND-diet. This material systematically highlights key brain-protective and gut-friendly components, such as the frequent use of extra virgin olive oil, legumes (e.g., lentils, beans), nuts, berries, and whole grains (including less commonly consumed grains or pseudograins such as quinoa and spelt). Both groups will be provided with recipe examples to support their adherence to a healthy diet. Additionally, all participants will be advised to limit their alcohol consumption to 2–3 servings per week, avoid sodas (both sugared and artificially sweetened), and prioritize water as their primary beverage. This approach aligns with the concept of an “active” control group, which is widely considered best practice (14). Providing the control group with general dietary advice helps ensure that any observed differences between the intervention and control group are more likely due to the specific characteristics of the MIND diet rather than the simple act of offering structured dietary guidance. While some confounding may arise from differences in how dietary advice is communicated, we believe our strategy minimizes these effects, allowing for a more precise assessment of the MIND diet's impact. To minimize possible bias, we will encourage participants not to share any details of their dietary guidance or meal plans with other participants enrolled in this study. We also acknowledge that the control group may improve their eating habits due to participation in the study, for example, by increasing their consumption of foods traditionally considered healthy, such as fruits and vegetables.

The MIND diet combines elements from the Mediterranean and DASH diets to enhance brain health. It emphasizes nutrient-dense plant-based foods, such as leafy greens and berries, which are rich in antioxidants. Healthy fats, such as omega-3 fatty acids found in olive oil and nuts, support cognitive function (6, 15). Whole grains are preferred for their high fiber content, which aids in blood sugar control and provides sustained energy. Lean proteins, such as fish and poultry, which are low in saturated fats and high in polyunsaturated fats, are encouraged over red meat. The diet promotes moderation in sodium intake and advises limiting sweets and unhealthy fats to maintain cardiovascular health. Overall, the MIND diet aims to optimize brain health through a balanced and nutritious eating pattern and is associated with better cognitive performance and brain structure in people with obesity (6, 15).

#### 2.3.3 Criteria for discontinuing or modifying the intervention

Participation in the MIND-GUT study may be interrupted or discontinued in the event of unanticipated side effects or health issues (e.g., fatigue, gastrointestinal disturbances, and nausea).

#### 2.3.4 Endpoints

The primary endpoints of this trial are measures of feasibility and acceptability of the intervention. Feasibility will be evaluated by retention (completion rates) and adherence to the diet protocol. The acceptability of the intervention will be evaluated by identifying themes emerging in qualitative interviews or another appropriate qualitative method.

The key secondary endpoints are the percent change changes in anthropometry (mainly body weight, BMI, and waist and hip circumferences), and mental wellbeing (i.e., perceived stress, depression, and anxiety symptoms) over 12 weeks, as well as alterations in the gut microbiota composition and diversity at baseline and 12 weeks. By examining these various endpoints, the study will provide an assessment of the intervention's effects on both physical and mental health (see Table 1).

#### 2.3.5 Assignment of intervention and control groups

We will implement a stratified block randomization approach to ensure balanced allocation of participants by sex. Initially, participants will be stratified into two groups based on biological sex. Within these strata, block randomization will generate randomization lists with predefined block sizes, ensuring an equal distribution across the intervention and control groups. Unique participant IDs will be generated and randomized separately by sex. This method maintains randomization integrity and balance, accommodating the staged and uneven recruitment anticipated.

#### 2.3.6 Stool samples and microbiota data

Participants will be asked to provide a stool sample at enrollment, as well as at 6 and 12 weeks, using suitable collection kits. Collecting multiple samples (even more than three as in this study) is recommended by the majority of researchers in microbiota research, since latter is intrinsically dynamic (16). Stool samples will be self-collected by participants using a provided collection device equipped with a small spoon, which will then be securely sealed in a sterile vial. The samples will be stored at −20 °C (home freezer) until submission to the research team. Participants will be instructed to collect the stools after urinating, using disposable paper taped inverted over the toilet to form a collection area that prevents contact with both skin and water. Participants will be instructed to collect a stool sample only if they have not had a fever, gastroenteritis, or diarrhea within the past 24 h. They should refrain from taking the test if they have undergone antibiotic treatment or experienced diarrohea in the previous week. They will also complete a questionnaire to collect information on stool features, including the Bristol stool chart (16). At our facility, the samples will be kept frozen at −20 °C until the analyses. Finally, to minimize batch effects during laboratory processing, DNA extraction will be performed in a randomized order and will include appropriate negative and positive controls. Table 3 lists the main variables collected in this study.

**Table 3:** Measures among intervention and control groups.

**Variables**	**Baseline/Follow-up**
**Sociodemographic**	
Date of birth	Baseline only
Biological sex at birth	
Educational level attained	
Marital status	
15.6-2.2,-1.3242ptRecruitment location	
**Anthropometry**	
Body height	Baseline and follow-up
Body weight	
15.6-2.2,-1.3242ptWaist & hip circumferences	
**Biological**	
Systolic and diastolic blood pressure	Baseline and follow-up
Stool collection	
Bowel movements (frequency)	
Irritable bowel symptoms	
15.6-2.2,-10.3242ptMenopause/Time since last menstrual period (LMP)	
**Body image**	
Stunkard Scale for body image	Baseline and follow-up
15.6-2.2,-1.3242ptEating Attitudes Test (EAT-26)	
**Mental wellbeing**	
PHQ-9 score for depressive symptoms	Baseline and follow-up
PSS-4 score for perceived stress	
GAD-7 score for anxiety symptoms	
15.6-2.2,-1.3242ptUsual sleep duration/24 h	
**Other variables**	
WHO physical activity questionnaire	Baseline and follow-up
Prescription medications	
Smoking status and use of e-cigarettes or chewing tobacco (snus)	

EAT, Eating Attitude Test; PHQ, Participants Health Questionnaire; PSS-4, Perceived Stress Score-4; GAD, Generalized Anxiety Disorders; WHO, World Health Organization.

#### 2.3.7 Study validity and reliability

To minimize observer bias, we will provide extensive training to the staff involved in the study. Interviewers will receive comprehensive training on interview techniques, including active listening, non-leading questioning, and maintaining a neutral stance. Standardized interview protocols will be followed, and regular supervision and feedback sessions will be conducted to monitor and address any potential biases that may arise. Clear and specific instructions will be provided to participants to minimize recall bias. Additionally, we will emphasize the confidentiality and anonymity of participant responses to reduce social desirability bias. To ensure reliable and repeatable study results, we will establish standardized protocols for all procedures, including participant recruitment, data collection, intervention delivery, and outcome assessments. Robust data management systems will be implemented to ensure accurate data collection, entry, and storage, with regular quality checks to identify and rectify any discrepancies.

### 2.4 Statistical and protocol analyses

#### 2.4.1 Definition and analysis of primary endpoints (feasibility and adherence)

Retention will be defined as the proportion of enrolled participants who completed the 12-week protocol. This will be calculated for the entire sample and stratified by sex at birth. Specific analyses will be conducted to examine adherence in relation to the variables measured at recruitment, particularly BMI and mental wellbeing (i.e., perceived stress, depression, and anxiety symptoms).

Adherence to the intervention diet will be analyzed by comparing the average frequencies and portion sizes of food reported via food checklists at baseline, 6, and 12 weeks with the principles and recommendations outlined in the MIND diet (8). For portions reported in volumes, intakes in grams will be estimated. Deviations from recommended intake frequencies will be calculated to define levels of dietary compliance. Participants reporting >80% adherence will be considered fully compliant; those with 60%−79% adherence, partially compliant; 40%−59%, partially non-compliant; and < 40%, non-compliant. Non-adherence or loss to follow-up will be dealt with by supplementing the primary analyses [conducted based on the “intention-to-treat” (ITT) principle] with additional methods, such as “per protocol” and “as treated” analyses (17). Trial results will be communicated via email to participants and stakeholders and disseminated through publications and results databases.

#### 2.4.2 Planned statistical analyses of secondary and exploratory outcomes

Baseline anthropometry and mental wellbeing characteristics will be presented and compared between groups using *t*-tests and χ^2^-tests based on the intention-to-treat principle. Retention will be analyzed by comparing completion rates between groups.

Missing data will be handled using a Mixed Model for Repeated Measures approach as the primary method, complemented by Multiple Imputation sensitivity analyses. Secondary anthropometric, mental wellbeing, and gut microbiome outcomes will be analyzed using repeated measures ANOVA and Linear Mixed Effects Models (per protocol and as-treated). Mixed models will include Participant ID as a random effect to account for within-subject correlation. Fixed effects will include treatment group and key covariates (age, baseline values of outcome variables). Sex will be included as a fixed effect to test for Sex × Treatment interactions; if significant, analyses will be stratified by sex. Sensitivity analyses will also explicitly test the interaction with and adjust for country of birth. Additional potential confounders and effect modifiers (baseline BMI, smoking status, and physical activity) will be added stepwise and retained if statistically significant.

Model assumptions will be assessed prior to analysis. For continuous outcomes, normality will be evaluated using Q-Q plots, histograms, and Shapiro–Wilk tests. Residuals will be examined for normality and homoscedasticity. Non-normal continuous variables will be transformed (e.g., logarithmic transformation) as appropriate.

For gut microbiota, differential abundance and diversity will be analyzed using compositional methods (e.g., ANCOM-BC or DESeq2), observed features, Shannon index, and beta diversity metrics. The False Discovery Rate will be controlled for multiple comparisons. Sensitivity analyses will adjust for country of birth. Given the exploratory nature of this trial, significance will be set at α = 0.10. Interim analyses will be performed at set intervals by the lead researcher and collaborators.

### 2.5 Ethics and dissemination

This study has been approved by the Swedish Ethical Authority (Dnr 2023-05478-01). Informed consent will be obtained from all participants before their participation. Results will be disseminated through peer-reviewed publications and conferences.

## 3 Discussion

Despite extensive research, obesity remains a challenging condition to treat (18). Adults with obesity face higher risks of depression, stress (19), and chronic disease. While the direction of the relationship between obesity and mental health remains unclear (20), it appears to vary from individual to individual. The “jolly fat hypothesis” once suggested fewer depressive symptoms among those with obesity (21). Yet, evidence shows that mental health often worsens after bariatric surgery, with increased risks of depression and suicide (22). Repeated dieting failures and weight stigma—especially among women—further impact psychological wellbeing, contributing to a bidirectional link where distress fuels weight gain and obesity worsens mental health (23).

Pharmacological options exist for both mental health and obesity. In particular, GLP-1 receptor agonists such as liraglutide and semaglutide effectively support weight loss and appetite reduction, and show potential benefits for mental wellbeing (24). Unfortunately, they are expensive, not always available to patients with obesity, and side effects have been observed (25). Additionally, as soon as treatment is stopped, patients generally regain the lost weight within 1 year (26). Therefore, the availability of pharmacological treatments does not negate the positive impacts of lifestyle therapies (particularly diet), as a cornerstone of obesity management, which are endorsed by international guidelines (27).

Dietary interventions have shown potential to positively modify the gut microbiota, thereby improving both physical and mental health outcomes. Considering the individual-specific nature of microbiota responses to nutritional changes, it is reasonable to assume that nutrition advice guided by microbiota analysis holds the potential to transform body weight loss interventions through personalized nutrition advice (28). A Western diet—high in processed foods and fat, and low in fiber—can induce dysbiosis, impairing both metabolic and mental health (29). In contrast, short-chain fatty acids produced from fiber fermentation support insulin sensitivity and energy regulation (30). Lower levels of Bifidobacterium are associated with higher depression risk and may contribute to weight dysregulation via effects on gut barrier integrity and metabolism (31).

This study has several strengths, including the implementation of standardized protocols for participant recruitment, data collection, intervention delivery, and outcome assessments. By ensuring consistency across all study sites, we aim to reduce variability and enhance the reliability of our findings. Furthermore, extensive training for interviewers and research assistants on interview techniques and the use of standardized interview protocols helps minimize observer bias. A robust data management system, including regular data quality checks and oversight by a data monitoring committee, will ensure the integrity and accuracy of the data collected. Comprehensive documentation of our research methodology, including study design, sample characteristics, intervention protocols, and outcome assessment procedures, facilitates the reproducibility of our study and enables other researchers to validate our findings independently.

Despite its strengths, the study has limitations that must be acknowledged. The choice of primarily recruiting participants based on obesity rather than the presence of clinically significant mental health conditions could capture a high proportion of subsyndromal symptoms, not severe enough to demonstrate meaningful change within the intervention period. However, psychiatric and metabolic medications commonly prescribed in such populations (e.g., antidepressants, antipsychotics, metformin) have well-documented effects on the gut microbiota and metabolic pathways, which would substantially confound the interpretation of dietary effects (32, 33). Moreover, studies consistently show that individuals with psychiatric disorders face greater challenges in recruitment, adherence, and compliance with dietary interventions, particularly in longer-term trials (34). Given that this was the first implementation of our study design in an obese population, we considered it methodologically sound to first establish feasibility and effect sizes in a non-clinical sample, minimizing confounding from psychotropic medication and illness severity.

Another limitation is that potential biases, such as selection bias, recall bias, and social desirability bias, could affect the validity of our findings. To mitigate these, we will provide clear instructions to participants and emphasize the confidentiality and anonymity of their responses. Another limitation is the reliance on self-reported dietary data, which may introduce inaccuracies due to misreporting or recall bias due to issues with memory recall. To reduce these, we utilize a food frequency checklist administered at three distinct time points (recruitment, 6 weeks, and 12 weeks) to track adherence over time. Furthermore, as is often inherent in non-placebo-controlled dietary trials, the risk of participants determining their group allocation (unblinding) remains a possibility due to the noticeable difference in the diet guidance and recipe books provided. Finally, the intervention's duration may not be sufficient to observe long-term effects; therefore we plan to implement follow-up surveys. The insights gained from this study will also provide valuable information to inform the design of larger studies.
