# Supplementary material for: Exploring the interplay between diet, obesity, mental health, and the gut microbiota: the MIND-GUT intervention study, study protocol
Source: Front Nutr. 2025 Dec 10;12:1703255. doi: 10.3389/fnut.2025.1703255 (PMC12731245; doi:10.3389/fnut.2025.1703255)
Supplement: Supplementary file 1 [file Table_1.docx]

**SUPPLEMENTARY MATERIALS**

**Questionnaires**

**General questionnaire**

Please complete the following questionnaire to provide information for the main study:

Participant ID:

**General questions**

**What is your date of birth?**

__/__/____

**In which country were you born?**

- Sweden
- Other (please specify: ___________)

**Are you:**

- Married/Registered partnership
- Single
- Divorced
- Widow(er)

**What is your biological Sex at Birth?**

- Male
- Female
- Other (please specify: ___________)

**With what gender do you identify?**

- Male
- Female
- Non-binary
- Other (please specify: ___________)

**Do you smoke?**

- No, I have never smoked (or smoked less than 100 cigarettes in my whole life)
- Yes, I have smoked more than 100 cigarettes in my life, but I have stopped now
- Yes and I still smoke only regular cigarettes
- Yes, but I only smoke electronic cigarettes
- Yes and I smoke both regular and electronic cigarettes

**How many regular cigarettes do you smoke every day (or used to smoke in case you stopped)?**

- 0-2 cigarettes per day
- 3-5 cigarettes per day
- 6-10 cigarettes per day
- 11-20 cigarettes per day
- More than 20 cigarettes per day

**On what year have you stopped smoking at latest? Reply only if you used to smoke.**

____

**Do you use snus?**

- Yes
- No

**If yes, how long have you used snus?**

- Less than 6 months
- 6 months to 1 year
- 1 to 3 years
- More than 3 years

**How often do you use snus?**

- A few times during the week
- Once a day
- 2-3 times a day
- 4-5 times a day
- More than 5 times a day

**What is your highest grade?**

- I have no school grades/I have not completed primary school
- Primary school
- Intermediate school
- Lower secondary school
- Upper secondary school
- Vocational school
- Bachelor degree
- Master degree
- Phd/Doctorate
- Other

**What is your main occupation?**

- I am unemployed
- I have my own company
- I work for a private company
- I work for a public/state organization
- I am a pensioner
- I am a student

**Questions on health perception**

**Sleep health**

**On average, how many hours of sleep do you get per night?**

- Less than 5 hours
- 5-6 hours
- 6-7 hours
- 7-8 hours
- More than 8 hours

**Current medications**

**Are you taking any medication that your doctor prescribed to you?**

- Yes
- no

**If you have answered “yes” to the previous question, please list the medications you are taking below, together with the health condition for which they were prescribed and the prescribed dose.**

**Medication 1 (name)**

___________________

**Health condition/disease for which it was prescribed**

___________________

**Dos (e.g., mg)**

___________________

**How many times a time do you take this medication?**

____

**Medication 2 (name)**

___________________

**Health condition/disease for which it was prescribed**

___________________

**Dos (e.g., mg)**

___________________

**How many times a time do you take this medication?**

____

**Medication 3 (name)**

___________________

**Health condition/disease for which it was prescribed**

___________________

**Dos (e.g., mg)**

___________________

**How many times a time do you take this medication?**

____

**Medication 4 (name)**

___________________

**Health condition/disease for which it was prescribed**

___________________

**Dos (e.g., mg)**

___________________

**How many times a time do you take this medication?**

____

**Medication 5 (name)**

___________________

**Health condition/disease for which it was prescribed**

___________________

**Dos (e.g., mg)**

___________________

**How many times a time do you take this medication?**

____

**Questions on your gut health**

**How often do you typically have bowel movements?**

- Less than once a day
- Once a day
- More than once a day

**Please indicate the severity of the following symptoms experienced in the past month:**

Abdominal pain or discomfort:

- None
- Mild
- Moderate
- Severe

Bloating or distension:

- None
- Mild
- Moderate
- Severe

Changes in bowel habits (e.g., diarrhea, constipation):

- None
- Mild
- Moderate
- Severe

**Other questions**

**How much time has passed since your last period (women only)**: __________

**How would you describe your current menstrual status?**

- I have regular periods
- Perimenopause/Menopause transition (changes in periods, but have not gone 12 months in a row without a period)
- Natural menopause
- Surgical menopause
- Other (explain):________________________

**Body Rating Scale:**

On a scale of 1-10, please rate your current body satisfaction (1 = extremely dissatisfied, 10 = extremely satisfied)?

____________

**Look at the following picture and then answer the two questions below.**


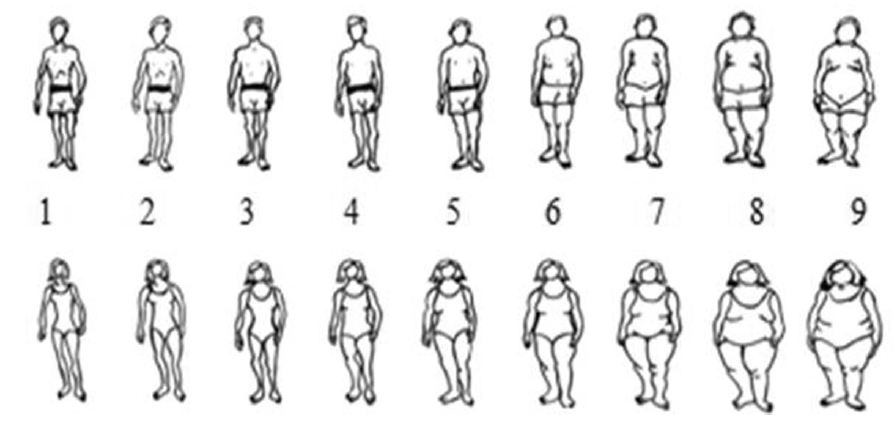


1. Which silhouette looks most like you?
   - 1
   - 2
   - 3
   - 4
   - 5
   - 6
   - 7
   - 8
   - 9
2. Which silhouette would you like to look like?
   - 1
   - 2
   - 3
   - 4
   - 5
   - 6
   - 7
   - 8
   - 9

**Is there anything else you would like to add or specify? Use the space below to write your thoughts.**

Thank you for completing our questionnaire!

**GPAQ-Physical activity**

| **Physical Activity** | | | | | | | | | |
| --- | --- | --- | --- | --- | --- | --- | --- | --- | --- |
| Next I am going to ask you about the time you spend doing different types of physical activity in a typical week. Please answer these questions even if you do not consider yourself to be a physically active person.  Think first about the time you spend doing work. Think of work as the things that you have to do such as paid or unpaid work, study/training, household chores, harvesting food/crops, fishing or hunting for food, seeking employment. *[Insert other examples if needed].* In answering the following questions 'vigorous-intensity activities' are activities that require hard physical effort and cause large increases in breathing or heart rate, 'moderate-intensity activities' are activities that require moderate physical effort and cause small increases in breathing or heart rate. | | | | | | | | | |
| **Questions** | | | **Response** | | | | | | |
| **Activity at work** | | | | | | | | | |
| 1 | | Does your work involve vigorous-intensity activity that causes large increases in breathing or heart rate like *carrying or lifting* *heavy loads, digging or construction work* for at least 10 minutes continuously? *e.g., carrying or lifting heavy loads, digging or construction work, commercial fishing, search and rescue operations.* | Yes | | | | 1 | | |
|  |  |  | No | | | | 2  *If No, go to P 4* | | |
| 2 | | In a typical week, on how many days do you do vigorous-intensity activities as part of your work? | Number of days | | | | └─┘ | | |
| 3 | | How much time do you spend doing vigorous-intensity activities at work on a typical day? | Hours : minutes | | | | └─┴─┘: └─┴─┘  hrs mins | | |
| 4 | | Does your work involve moderate-intensity activity that causes small increases in breathing or heart rate such as brisk walking *or carrying light loads* for at least 10 minutes continuously? | Yes | | 1 | | | | |
|  |  |  | No | | 2 *If No, go to P 7* | | | | |
| 5 | | In a typical week, on how many days do you do moderate-intensity activities as part of your work? | Number of days | | └─┘ | | | | |
| 6 | | How much time do you spend doing moderate-intensity activities at work on a typical day? | Hours : minutes | | └─┴─┘: └─┴─┘  hrs mins | | | | |
| **Travel to and from places** | | | | | | | | | |
| The next questions exclude the physical activities at work that you have already mentioned.  Now I would like to ask you about the usual way you travel to and from places. For example, to work, for shopping, to market, to place of worship. | | | | | | | | | |
| 7 | | Do you walk or use a bicycle (*pedal cycle*) for at least 10 minutes continuously to get to and from places? | Yes | 1 | | | | | |
|  |  |  | No | 2  *If No, go to P 10* | | | | | |
| 8 | | In a typical week, on how many days do you walk or bicycle for at least 10 minutes continuously to get to and from places? | Number of days | └─┘ | | | | | |
| 9 | | How much time do you spend walking or bicycling for travel on a typical day? | Hours : minutes | └─┴─┘: └─┴─┘  hrs mins | | | | | |
| **Recreational activities** | | | | | | | | | |
| The next questions exclude the work and transport activities that you have already mentioned.  Now I would like to ask you about sports, fitness, and recreational activities (leisure). | | | | | | | | | |
| 10 | | Do you do any vigorous-intensity sports, fitness or recreational (*leisure*) activities that cause large increases in breathing or heart rate like (e.g., *running or football)* for at least 10 minutes continuously? | Yes | | | 1 | | | |
|  |  |  | No | | | 2  *If No, go to P 13* | | | |
| 11 | | In a typical week, on how many days do you do vigorous-intensity sports, fitness or recreational (*leisure*) activities? | Number of days | | | └─┘ | | | |
| 12 | | How much time do you spend doing vigorous-intensity sports, fitness or recreational activities on a typical day? | Hours : minutes | | | └─┴─┘: └─┴─┘  hrs mins | | | |
| **Physical Activity (recreational activities)** contd. | | | | | | | | |  |
| **Questions** | | **Response** | | | | | | |  |
| 13 | | Do you do any moderate-intensity sports, fitness or recreational *(leisure*) activities that causes a small increase in breathing or heart rate such as brisk walking (e.g., *cycling, swimming, volleyball*) for at least 10 minutes continuously? | Yes | | | | | 1 | |
|  |  |  | No | | | | | 2  *If No, go to P16* | |
| 14 | | In a typical week, on how many days do you do moderate-intensity sports, fitness or recreational (*leisure*) activities? | Number of days | | | | | └─┘ | |
| 15 | | How much time do you spend doing moderate-intensity sports, fitness or recreational (*leisure*) activities on a typical day? | Hours : minutes | | | | | └─┴─┘: └─┴─┘  hrs mins | |
| **Sedentary behaviour** | | | | | | | | | |
| The following question is about sitting or reclining at work, at home, getting to and from places, or with friends including time spent (e.g., sitting at a desk, sitting with friends, travelling in car, bus, train, reading, playing cards, or watching television), but do not include time spent sleeping. | | | | | | | | | |
| 16 | | How much time do you usually spend sitting or reclining on a typical day? | Hours : minutes | | | | | | └─┴─┘: └─┴─┘  hrs min s |

**Eating Attitude Test (EAT-26)**

| Instructions: This is a screening measure to help you determine whether you might have an eating disorder that needs professional attention. This screening measure is not designed to make a diagnosis of an eating disorder or take the place of a professional consultation. Please fill out the below form as accurately, honestly and completely as possible. There are no right or wrong answers. All of your responses are confidential. | | | | | | | | | | | | | | | | | | | | | | | | | | | | | | |  |
| --- | --- | --- | --- | --- | --- | --- | --- | --- | --- | --- | --- | --- | --- | --- | --- | --- | --- | --- | --- | --- | --- | --- | --- | --- | --- | --- | --- | --- | --- | --- | --- |
| **Part A: Complete the following questions:** | | | | | | | | | | | | | | | | | | | | | | | | | | | | | | |  |
| 1) Birth Date | | | Month: | |  | Day: |  | Year: | |  | | 2) Sex: | | | | | | Male | | | | Female | | | | |  | | |  |  |
| 3) Height Feet : | | | |  | Inches: |  |  |  | |  | | | | | | | | □ | | | | □ | | | | |  | | |  |  |
| 4) Current Weight (lbs.): | | | | |  | 5) Highest Weight (excluding pregnancy): | | | | | | | | |  | | | | | |  | | | | | | | | |  |  |
| 6) Lowest Adult Weight: | | | | |  | 7: Ideal Weight: | | |  | |  | | | | | | | | | | | | | | | | | | |  |  |
| **Part B: Check a response for each of the following statements:** | | | | | | | | | | | | | | Always | | | Usually | | | Often | | | | Some  times | | Rarely | | | Never | | |
| 1. | | Am terrified about being overweight. | | | | | | | | | | | | □ | | | □ | | | □ | | | | □ | | □ | | | □ | | |
| 2. | | Avoid eating when I am hungry. | | | | | | | | | | | | □ | | | □ | | | □ | | | | □ | | □ | | | □ | | |
| 3. | | Find myself preoccupied with food. | | | | | | | | | | | | □ | | | □ | | | □ | | | | □ | | □ | | | □ | | |
| 4. | | Have gone on eating binges where I feel that I may not be able to stop. | | | | | | | | | | | | □ | | | □ | | | □ | | | | □ | | □ | | | □ | | |
| 5. | | Cut my food into small pieces. | | | | | | | | | | | | □ | | | □ | | | □ | | | | □ | | □ | | | □ | | |
| 6. | | Aware of the calorie content of foods that I eat. | | | | | | | | | | | | □ | | | □ | | | □ | | | | □ | | □ | | | □ | | |
| 7. | | Particularly avoid food with a high carbohydrate content (i.e. bread, rice, potatoes, etc.) | | | | | | | | | | | | □ | | | □ | | | □ | | | | □ | | □ | | | □ | | |
| 8. | | Feel that others would prefer if I ate more. | | | | | | | | | | | | □ | | | □ | | | □ | | | | □ | | □ | | | □ | | |
| 9. | | Vomit after I have eaten. | | | | | | | | | | | | □ | | | □ | | | □ | | | | □ | | □ | | | □ | | |
| 10. | | Feel extremely guilty after eating. | | | | | | | | | | | | □ | | | □ | | | □ | | | | □ | | □ | | | □ | | |
| 11. | | Am preoccupied with a desire to be thinner. | | | | | | | | | | | | □ | | | □ | | | □ | | | | □ | | □ | | | □ | | |
| 12. | | Think about burning up calories when I exercise. | | | | | | | | | | | | □ | | | □ | | | □ | | | | □ | | □ | | | □ | | |
| 13. | | Other people think that I am too thin. | | | | | | | | | | | | □ | | | □ | | | □ | | | | □ | | □ | | | □ | | |
| 14. | | Am preoccupied with the thought of having fat on my body. | | | | | | | | | | | | □ | | | □ | | | □ | | | | □ | | □ | | | □ | | |
| 15. | | Take longer than others to eat my meals. | | | | | | | | | | | | □ | | | □ | | | □ | | | | □ | | □ | | | □ | | |
| 16. | | Avoid foods with sugar in them. | | | | | | | | | | | | □ | | | □ | | | □ | | | | □ | | □ | | | □ | | |
| 17. | | Eat diet foods. | | | | | | | | | | | | □ | | | □ | | | □ | | | | □ | | □ | | | □ | | |
| 18. | | Feel that food controls my life. | | | | | | | | | | | | □ | | | □ | | | □ | | | | □ | | □ | | | □ | | |
| 19. | | Display self-control around food. | | | | | | | | | | | | □ | | | □ | | | □ | | | | □ | | □ | | | □ | | |
| 20. | | Feel that others pressure me to eat. | | | | | | | | | | | | □ | | | □ | | | □ | | | | □ | | □ | | | □ | | |
| 21. | | Give too much time and thought to food. | | | | | | | | | | | | □ | | | □ | | | □ | | | | □ | | □ | | | □ | | |
| 22. | | Feel uncomfortable after eating sweets. | | | | | | | | | | | | □ | | | □ | | | □ | | | | □ | | □ | | | □ | | |
| 23. | | Engage in dieting behavior. | | | | | | | | | | | | □ | | | □ | | | □ | | | | □ | | □ | | | □ | | |
| 24. | | Like my stomach to be empty. | | | | | | | | | | | | □ | | | □ | | | □ | | | | □ | | □ | | | □ | | |
| 25. | | Have the impulse to vomit after meals. | | | | | | | | | | | | □ | | | □ | | | □ | | | | □ | | □ | | | □ | | |
| 26. | | Enjoy trying new rich foods. | | | | | | | | | | | | □ | | | □ | | | □ | | | | □ | | □ | | | □ | | |
| **Part C: Behavioral Questions:**  **In the past 6 months have you:** | | | | | | | | | | | | | Never | | | Once a month or less | | | 2-3 times a month | | | | Once a week | | 2-6 times a week | | | Once a day or more | |  |  |
| A | Gone on eating binges where you feel that you may not be able to stop? * | | | | | | | | | | | | □ | | | □ | | | □ | | | | □ | | □ | | | □ | |  |  |
| B | Ever made yourself sick (vomited) to control your weight or shape? | | | | | | | | | | | | □ | | | □ | | | □ | | | | □ | | □ | | | □ | |  |  |
| C | Ever used laxatives, diet pills or diuretics (water pills) to control your weight or shape? | | | | | | | | | | | | □ | | | □ | | | □ | | | | □ | | □ | | | □ | |  |  |
| D | Exercised more than 60 minutes a day to lose or to control your weight? | | | | | | | | | | | | □ | | | □ | | | □ | | | | □ | | □ | | | □ | |  |  |
| E | Lost 20 pounds or more in the past 6 months | | | | | | | | | | | | Yes □ | | | | | | No □ | | | | | |  | | | | |  |  |
| ***** Defined as eating much more than most people would under the same circumstances and feeling that eating is out of control | | | | | | | | | | | | | | | | | | | | | | | | | | | | | |  |  |

**Patient Health Questionnaire (PHQ-9)**

ID# ___________________________________________________ DATE ____________________

Over the last 2 weeks, how often have you been bothered by any of the following problems?

|  | **Not at all** | **Several days** | **More than half the days** | **Nearly every day** |
| --- | --- | --- | --- | --- |
| 1. Little interest or pleasure in doing things |  |  |  |  |
| 1. Feeling down, depressed, or hopeless |  |  |  |  |
| 1. Trouble falling or staying asleep, or sleeping too much |  |  |  |  |
| 1. Feeling tired or having little energy |  |  |  |  |
| 1. Poor appetite or overeating |  |  |  |  |
| 1. Feeling bad about yourself or that you are a failure or have let yourself or your family down |  |  |  |  |
| 1. Trouble concentrating on things, such as reading the newspaper or watching television |  |  |  |  |
| 1. Moving or speaking so slowly that other people could have noticed. Or the opposite: being so figety or restless that you have been moving around a lot more than usual |  |  |  |  |
| 1. Thoughts that you would be better off dead, or of hurting yourself |  |  |  |  |

1. If you checked off any problems, how difficult have these problems made it for you to do your work, take care of things at home, or get along with other people?

**General Anxiety Disorder Questionnaire (GAD-7)**

| Over the last two weeks, how often have you been bothered by the following problems? | Not at all | Several days | More than half the days | Nearly every day |
| --- | --- | --- | --- | --- |
| 1. Feeling nervous, anxious, or on edge |  |  |  |  |
| 1. Not being able to stop or control worrying |  |  |  |  |
| 1. Worrying too much about different things |  |  |  |  |
| 1. Trouble relaxing |  |  |  |  |
| 1. Being so restless that it is hard to sit still |  |  |  |  |
| 1. Becoming easily annoyed or irritable |  |  |  |  |
| 1. Feeling afraid, as if something awful might happen |  |  |  |  |

If you checked any of the above problems, how difficult have they made it for you to do your work, take care of things at home, or get along with other people?

- Not difficult at all
- Somewhat difficult
- Very difficult
- Extremely difficult

**Perceived Stress Scale (PSS-4)**

**Instructions**

The questions in this scale ask about your feelings and thoughts during the past month. In each case, indicate your answer by selecting the box that represents how often you have felt or thought a certain way.

|  | **Never** | **Almost never** | **Sometimes** | **Quite often** | **Very often** |
| --- | --- | --- | --- | --- | --- |
| 1. In the past month, how often have you felt that you could not control important things in your life? |  |  |  |  |  |
| 1. In the past month, how often have you felt confident in your ability to handle personal problems? |  |  |  |  |  |
| 1. How often in the past month have you felt that things have gone your way? |  |  |  |  |  |
| 1. In the past month, how often have you felt that difficulties have piled up so much that you could not overcome them? |  |  |  |  |  |

**Consent form**

[*The text below has been translated from Swedish*]

**Consent to participate in a research project**

I have been given oral and/or written information about the study and have had the opportunity to ask questions. I may keep the written information.

- I agree to participate in the project «Exploring the interaction between diet, obesity, mental health and the gut microbiome. A digital intervention study.»
- I agree to my samples being stored in a biobank in the manner described in the research person information

| **Place and date** | **Signature** |
| --- | --- |
|  |  |
|  | **Printed Name** |
|  |  |
